# Supplementary material for: Fast and sensitive taxonomic classification for metagenomics with Kaiju
Source: Nat Commun. 2016 Apr 13;7:11257. doi: 10.1038/ncomms11257 (PMC4833860; doi:10.1038/ncomms11257)
Supplement: Supplementary Information — Supplementary Figures 1-6 and Supplementary Tables 1-2 [file ncomms11257-s1.pdf]

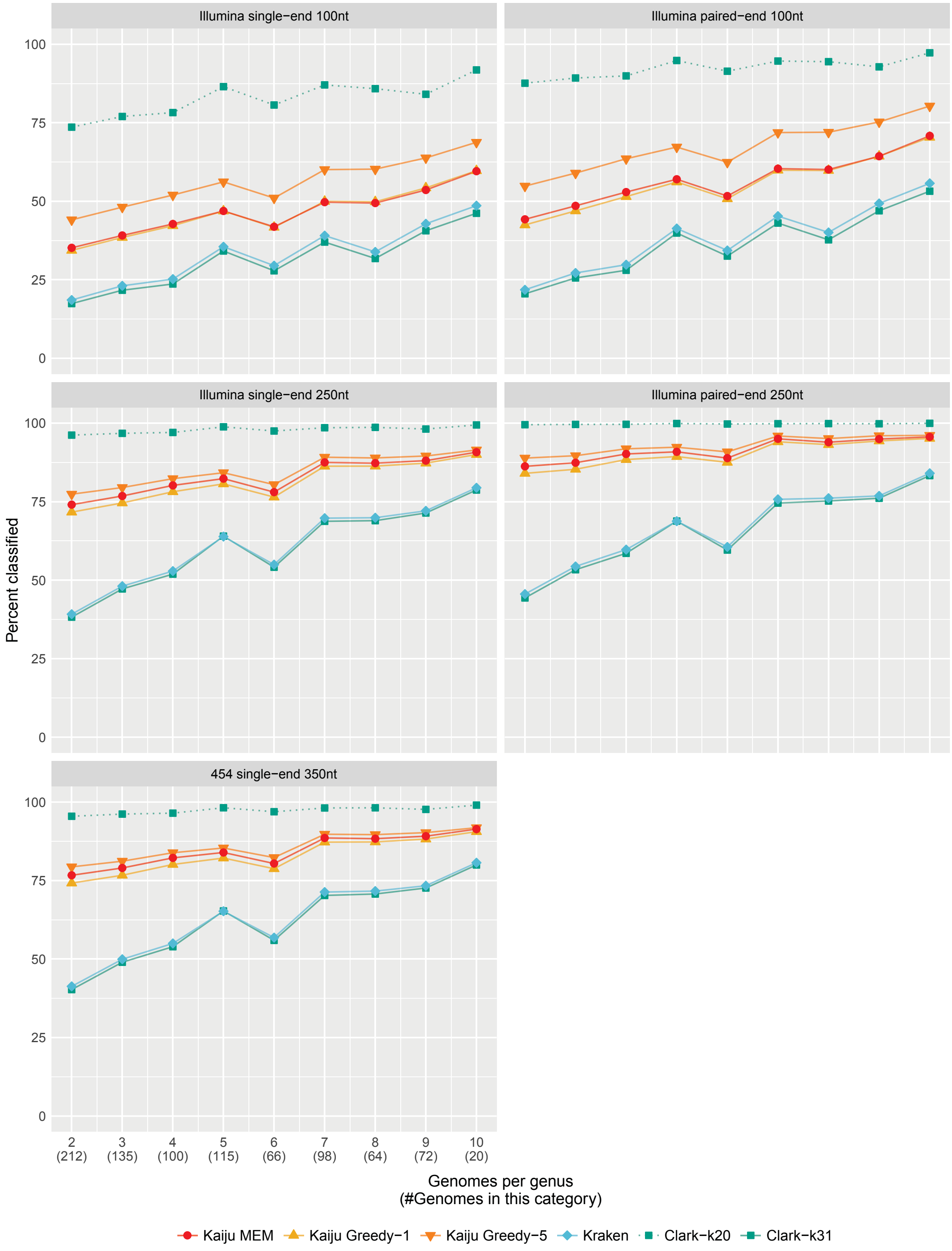

Suppl. Fig. 1: **Classified reads** Percentage of reads where classification was attempted by each program. Points denote the mean for each category of genera. The numbers for Clark were measured using the phylum-level index. Clark with $k = 20$ is denoted by the dotted line.

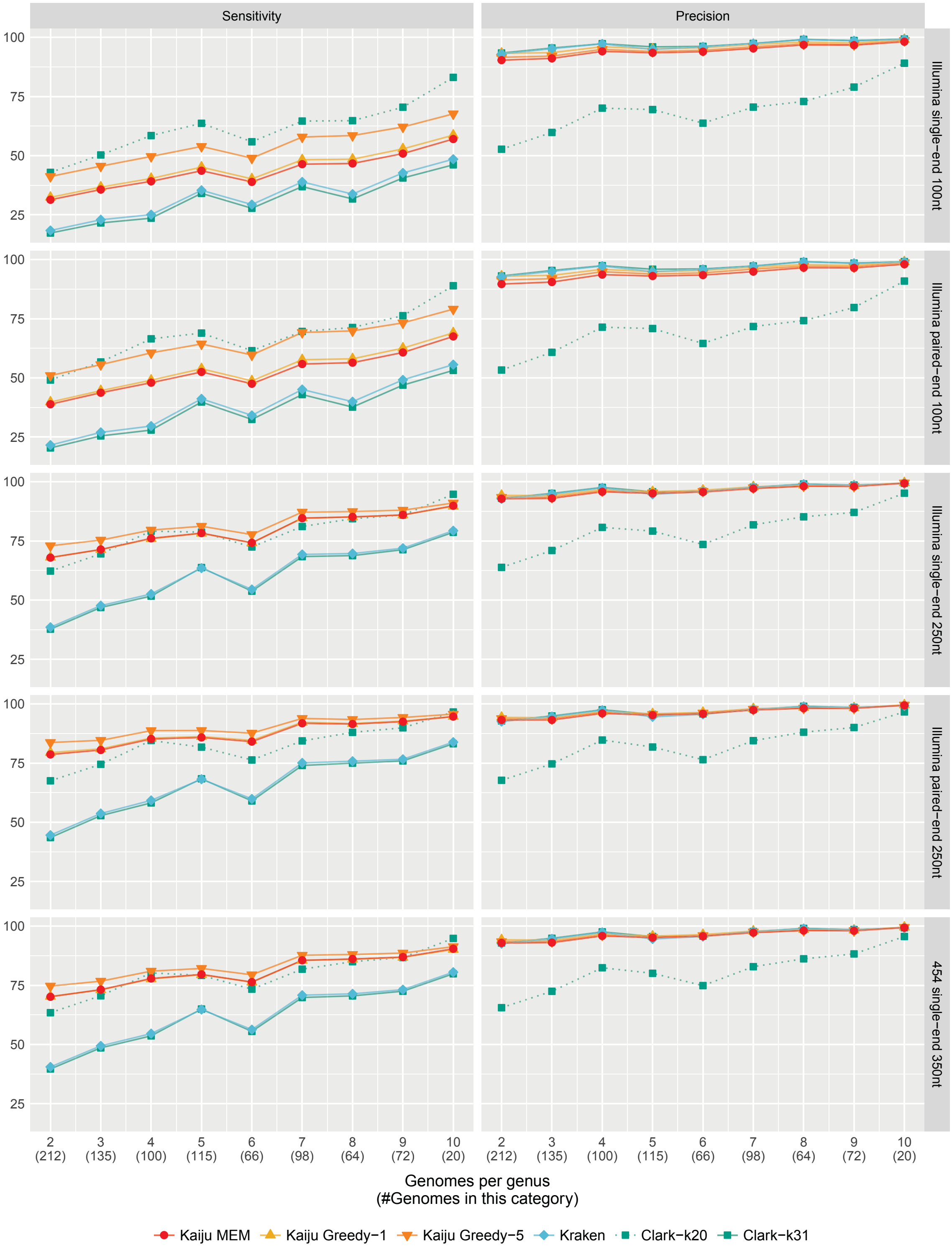

Suppl. Fig. 2: **Phylum-level sensitivity and precision** Sensitivity and precision were measured on phylum-level and are shown as average for each bin of genera for the five different types of reads and the three programs. Clark with $k = 20$ is denoted by the dotted line.

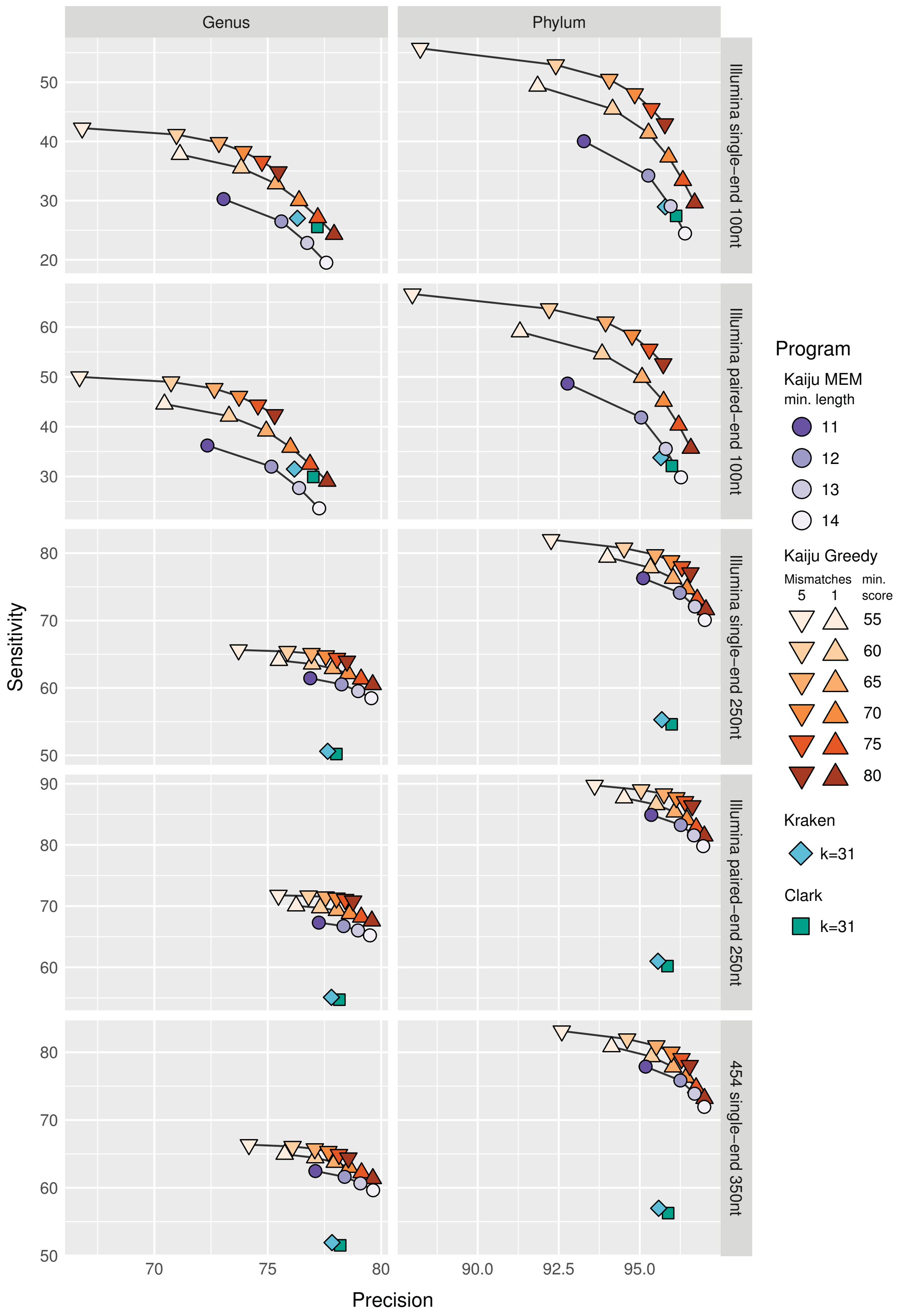

Suppl. Fig. 3: **Receiver operating characteristic** Genus-level sensitivity and precision for different values of minimum required match length m in Kaiju's MEM mode and minimum required match score s in Kaiju's Greedy mode. Points represent the mean across all 882 measured genomes in the benchmark.

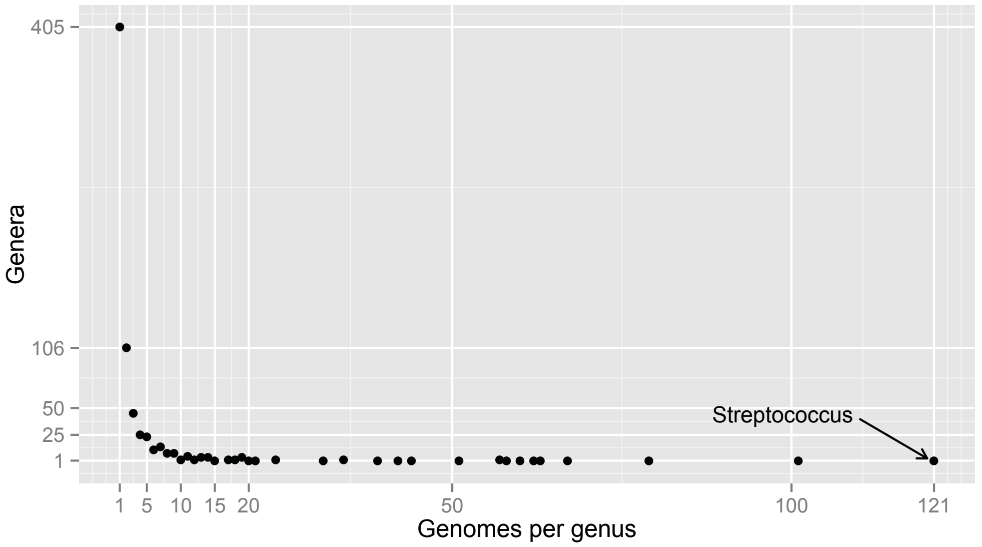

Suppl. Fig. 4: **Database composition** The figure illustrates the biased distribution of genomes to their respective genera in our snapshot of 2 724 archaeal and bacterial genomes from the NCBI database. For example, 121 genomes are available in the genus *Streptococcus*, whereas 405 genera have only one available genome.

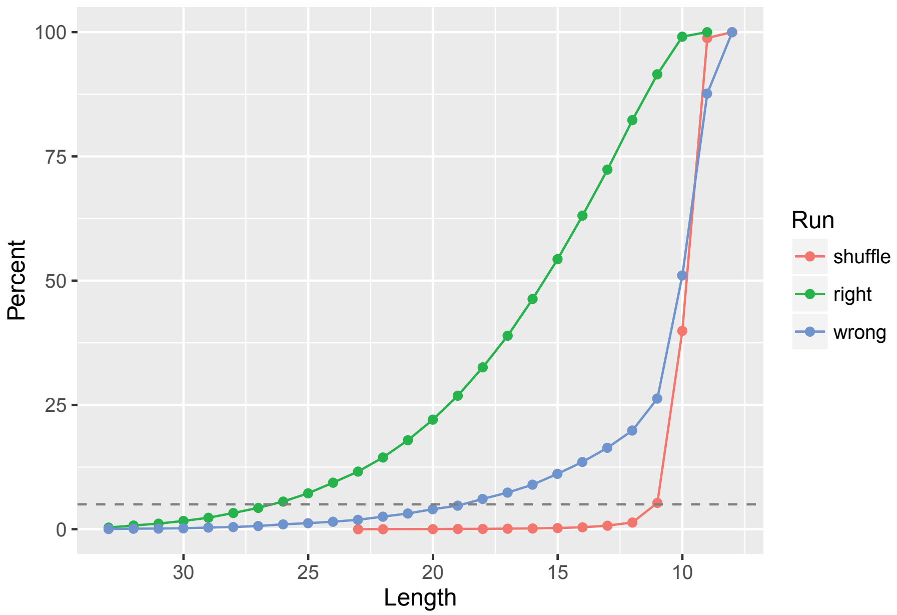

Suppl. Fig. 5: **Length distribution of MEMs** Distribution of match lengths for correctly (green) and incorrectly (blue) classified reads from a simulated mock metagenome. Lengths of random matches to a shuffled *NR* database are shown in red.

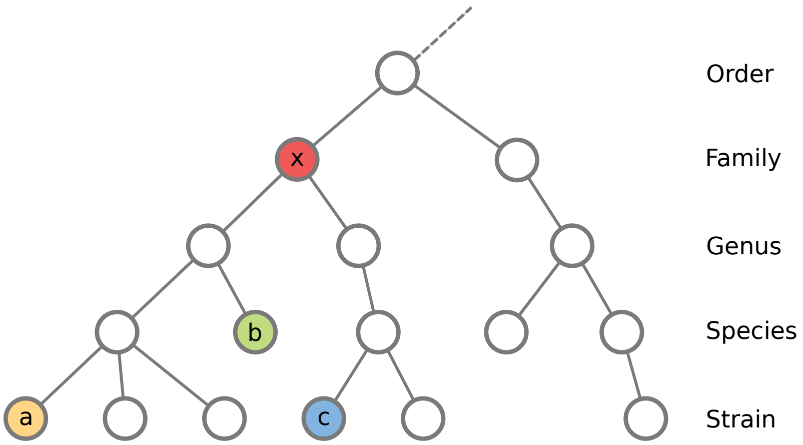

Suppl. Fig. 6: **Least Common Ancestor** If a sequencing read has matches with the same score to several different species/strains in the database, then Kaiju classifies the read to their least common ancestor (LCA) in the phylogenetic tree. The LCA can be found by traversing the tree upwards from the leaf nodes until all paths intersect. For example, the three leaves a, b, and c belong to two different genera so that their LCA is the node x directly above them on family level.

Suppl. Tab. 1: **Real metagenomes** Accession numbers from the Short Read Archive (`http://sra.dnanexus.com`) and read metadata of the ten real metagenomes. Run files from each sample were extracted using `fastq-dump` from the `sra-toolkit` using option `-E` for removing erroneous reads.

Name	SRA Acc nr	Instrument	Length	Total reads	Environment
Human Vagina	SRS015072	Illumina GA II	pe-100	699 174	human mid vagina
Human Saliva	SRS019120	Illumina GA II	pe-100	4 714 049	human saliva
Human Gut	SRS363878	Illumina MiSeq	pe-150	242 421	human stool from healthy individual
Cat Gut	SRS074452	454 GS FLX Tit.	42-2044	242 281	cat mid GI tract
Lake	SRS160185	Illumina GA II	pe-100	13 494 253	Lake Lanier freshwater
River Plume	SRS577849	Illumina MiSeq	pe-150	4 489 025	Amazon river plume surface seawater
Baltic Sea water	SRS291652	Illumina HiSeq	pe-150	29 391 416	Baltic Sea water 18-20.5m depth
Desert Soil	SRS445441	Ion Torrent PGM	8-315	2 420 832	desert and xeric shrubland
Bioreactor Sediment	SRR919301	Illumina MiSeq	pe-255	307 336	bioreactor inoculated with Wadden Sea sediment microbes
Bioreactor Compost	SRS009352	454 GS FLX	42-2044	538 591	bioreactor inoculated with switchgrass-adapted microbial community

Suppl. Tab. 2: **HiSeq and MiSeq mock communities** Sensitivity and precision measured on genus-level and phylum-level in the HiSeq and MiSeq datasets for Kraken ($k = 31$) and Kaiju's Greedy-5 mode ($s = 65$).

	HiSeq				MiSeq			
	Genus		Phylum		Genus		Phylum	
	Sens.	Prec.	Sens.	Prec.	Sens.	Prec.	Sens.	Prec.
Kraken	78.0	99.2	78.7	99.7	77.6	95.6	73.1	88.4
Greedy-5	73.3	94.4	78.1	98.3	72.1	90.0	81.0	89.5
